# Role of Acid Sphingomyelinase in the Regulation of Social Behavior and Memory

**DOI:** 10.1371/journal.pone.0162498

**Published:** 2016-09-06

**Authors:** Iulia Zoicas, Martin Reichel, Erich Gulbins, Johannes Kornhuber

**Affiliations:** 1 Department of Psychiatry and Psychotherapy, University Hospital, Friedrich-Alexander-University Erlangen-Nuremberg, Erlangen, Germany; 2 Department of Molecular Biology, University of Duisburg-Essen, Essen, Germany; Brock University, CANADA

## Abstract

Major depressive disorder is often associated with deficits in social and cognitive functioning. Mice transgenic for acid sphingomyelinase (t-ASM) were previously shown to have a depressive-like phenotype, which could be normalized by antidepressant treatment. Here, we investigated whether t-ASM mice show deficits in social behavior and memory performance, and whether these possible deficits might be normalized by amitriptyline treatment. Our results revealed that ASM overexpression altered the behavior of mice in a sex-dependent manner. As such, t-ASM female, but not male, mice showed an impaired social preference and a depressive- and anxiogenic-like phenotype, which could be normalized by amitriptyline treatment. Both male and female t-ASM mice showed unaltered preference for social novelty, novel object recognition, and social and object discrimination abilities. Amitriptyline treatment impaired novel object recognition and object discrimination abilities in female, but not in male, wild-type mice, while female t-ASM mice showed unaltered novel object recognition and object discrimination abilities. This study suggests that female t-ASM mice represent a model of depression with comorbid anxiety and social deficits, without memory impairments. It further suggests that ASM overexpression has a protective role against the detrimental effects of amitriptyline on female, but not on male, non-social (object) memory.

## Introduction

Major depressive disorder (MDD) is a severe and chronic mood disorder, with a lifetime prevalence of more than 10% [1]. Key symptoms of MDD are a depressed mood and loss of interest, anhedonia, feelings of worthlessness, weight loss, and insomnia. MDD is often associated with deficits in social functioning [2] and cognitive dysfunctions, such as memory impairment and concentration deficits [3].

Tricyclic antidepressant drugs, such as desipramine and imipramine, have been shown to induce the proteolytic degradation of the lysosomal glycoprotein acid sphingomyelinase (ASM) [4,5], an enzyme that catalyzes the hydrolysis of sphingomyelin into ceramide and phosphorylcholine [6], and thereby to functionally inhibit the activity of ASM [7]. These findings led to studies investigating the role of ASM in MDD and as a target mediating the effects of antidepressant drugs. As such, a clinical study found an increased ASM activity in peripheral blood mononuclear cells of patients experiencing a major depressive episode [8]. Transgenic mice overexpressing ASM (t-ASM) showed higher ASM activity and ceramide concentrations in the hippocampus, which were associated with a depressive- and anxiogenic-like phenotype as demonstrated in the novelty suppressed-feeding paradigm and in the open field test [9]. Amitriptyline, a tricyclic antidepressant, and fluoxetine, a selective serotonin reuptake inhibitor, have been shown to inhibit the activity of ASM, to reduce ceramide concentrations and ASM protein levels in cultured neurons and in the hippocampus of wild-type (WT) and t-ASM mice and to normalize the depressive- and anxiogenic-like phenotype of t-ASM mice when administered at doses that achieve therapeutic plasma concentrations recommended for patients with MDD [9]. In contrast, a genetic deficiency in ASM mimicked the effects of antidepressants and abrogated any further effects of antidepressants on depressive- and anxiety-like behavior in mice [9].

Considering the comorbidity between MDD, social deficits, and memory impairments, we aimed to investigate whether t-ASM mice show deficits in social behavior and memory performance and whether these possible deficits could be normalized by amitriptyline treatment. Given that depression is more prevalent in women and treatment response is often gender-dependent [10], we performed experiments in both male and female mice.

## Materials and Methods

### Animals

Mice conditionally overexpressing ASM were generated by a targeted integration of a murine *Smpd1* cDNA under the control of a cytomegalovirus (CMV) immediate early enhancer/chicken beta-actin fusion promoter (CAG) into the Hprt locus (Hprt^tm1.1(CAG-Smpd1)Jhkh^; www.informatics.jax.org/allele/MGI:5523896) [9]. A loxP-flanked STOP cassette between the promotor and the transgene prevented constitutive overexpression. Overexpression of ASM was initiated by crossing transgenic female mice with homozygous E2A-Cre male mice (Tg(EIIa-cre); http://www.informatics.jax.org/allele/MGI:2137691). Experiments were conducted with t-ASM and littermate WT controls from the F1 generation.

Male and female WT and t-ASM mice were individually housed for one week before treatment start and remained single-housed throughout the experiments. Age- and sex-matched WT mice were used as social stimuli for the social approach test. Sex-matched 3-week-old juvenile CD1 mice were used as social stimuli for the social discrimination test. Mice were kept under standard laboratory conditions (12:12 light/dark cycle, lights on at 06:00 h, 22°C, 60% humidity, food and water *ad libitum*). Experiments were performed during the light phase, between 09:00 and 14:00, in accordance with the recommendations in the Guide for the Care and Use of Laboratory Animals of the National Institutes of Health. The protocol was approved by the Committee on the Ethics of Animal Experiments of the Government of Mittelfranken (Permit Number: 54–2532.1-27/11). All efforts were made to minimize animal suffering and to reduce the number of animals used.

### Behavioral paradigms

Mice were tested in several behavioral tests and the order of the test sessions was selected to minimize any effect of one test session on subsequent sessions. As such, mice were tested in the social approach test on day 29, in the novel object recognition test on day 33, in the social discrimination test on day 37, in the object discrimination test on day 40, and in the novelty-suppressed feeding on day 45.

### Social approach test

The social preference and the preference for social novelty of mice were tested in the social approach test [11], with some minor modifications. Mice were placed in a novel arena (42 x 24 x 35 cm) and allowed to habituate for 30 s. Two identical wire-mesh cages (7 x 7 x 6 cm) were simultaneously placed at opposite side-walls of the arena for 5 min. One cage remained empty and one cage contained an unfamiliar age- and sex-matched conspecific (*same* mouse). The initial position of the *same* mouse varied between experimental mice to prevent for possible place preference. After 5 min, the empty cage was exchanged by an identical cage containing a *novel* mouse for additional 5 min. Experiments were recorded and the time spent investigating (sniffing) the empty cage, the *same* and the *novel* mouse was analyzed by an observer blind to the treatment condition using JWatcher (Version 1.0, Macquarie University and UCLA). The percentage of time investigating the empty cage and the *same* mouse (time investigating empty cage or *same* mouse/time investigating empty cage + *same* mouse x 100%) during the first 5 min, and the percentage of time investigating the *same* and the *novel* mouse (time investigating *same* or *novel* mouse/time investigating *same* + *novel* mouse x 100%) during the second 5 min was calculated. A higher investigation time directed toward the *same* mouse versus the empty cage during the first 5 min was interpreted as social preference. A higher investigation time directed toward the *novel* versus the *same* mouse during the second 5 min was interpreted as social recognition and preference for social novelty.

### Social discrimination test

The ability of mice to discriminate between a previously encountered (*same*) and a *novel* conspecific was tested in the social discrimination test as previously described [12,13]. A sex-matched juvenile mouse was introduced in the home cage of the experimental mouse for 4 min (acquisition period). After an interval of 60 min, the *same* juvenile was reintroduced along with a *novel* juvenile for additional 4 min (discrimination period). Experiments were recorded and the time spent investigating the juvenile mice (sniffing the anogenital and head/neck regions) was analyzed by an observer blind to the treatment condition using JWatcher. The percentage of time investigating the *same* and the *novel* juvenile mouse (time investigating *same* or *novel* mouse/time investigating *same* + *novel* mouse x 100%) during the discrimination period was calculated. A higher investigation time directed toward the *novel* versus the *same* juvenile was interpreted as social discrimination and intact social memory. The 3-week-old juvenile mice did not elicit play or aggressive behaviors towards the experimental mice.

### Novel object recognition test

The ability of mice to recognize novelty over familiarity was tested in the novel object recognition test [14], which was adapted to be comparable to the social approach test described above. Mice were placed in the arena (42 x 24 x 35 cm) and allowed to habituate for 30 s. Two identical objects (*same)* were simultaneously placed at opposite side-walls of the arena for 5 min. After 5 min, one object was exchanged by a *novel* object for additional 5 min. Several plastic objects that differed in color (pink, green, orange), shape (flower, diamond, square), and size (3–4.5 cm x 3 cm x 1 cm) were used. Objects were cleaned between trials with water containing a small amount of detergent (Manisoft; Ecolab Deutschland GmbH). Experiments were recorded and the time spent investigating the objects (sniffing/touching) was analyzed by an observer blind to the treatment condition using JWatcher. The percentage of time investigating the *same* and the *novel* object (time investigating *same* or *novel* object/time investigating *same* + *novel* object x 100%) during the second 5 min was calculated. A higher investigation time directed toward the *novel* versus the *same* object was interpreted as object recognition and preference for novelty.

### Object discrimination test

The ability of mice to discriminate between a previously encountered (*same*) and a *novel* object was tested in the object discrimination test [12,13], which was adapted to be comparable to the social discrimination test described above. An unknown object (*same*) was placed in one corner of the home cage of the experimental mouse for 4 min (acquisition period). The initial position of the object varied between mice to prevent for possible place preference. After an interval of 60 min, the *same* object was reintroduced along with a *novel* object for additional 4 min (discrimination period). The same objects were used as in the novel object recognition test described above. Experiments were recorded and the time spent investigating the objects (sniffing/touching) was analyzed by an observer blind to the treatment condition using JWatcher. The percentage of time investigating the *same* and the *novel* object (time investigating *same* or *novel* object/time investigating *same* + *novel* object x 100%) during the discrimination period was calculated. A higher investigation time directed toward the *novel* versus the *same* object was interpreted as object discrimination and intact object memory.

### Novelty-suppressed feeding paradigm

The depressive- and anxiety-like behavior of mice was tested in the novelty-suppressed feeding paradigm as previously described [9]. Mice were food-deprived for 24 h prior to testing with unlimited fluid access. Mice were placed in a novel arena (50 x 50 x 50 cm) with the head facing one of the corners. Immediately afterwards, a single food pellet (ssniff Spezialdiäten GmbH, Soest, Germany) was placed in the centre of the arena. The latency to feed, defined as biting the food pellet for longer than 3 s, was manually analyzed by an observer blind to the treatment condition. An increased feeding latency was interpreted as increased depressive- and anxiety-like behavior.

### Drugs

Amitriptyline (Amitriptyline hydrochloride, A8404; Sigma-Aldrich, Germany) was administered via drinking water at a dose of 180 mg/L, based on previous studies [9,15]. Treatment was started 4 weeks before and was maintained throughout behavioral testing.

### Statistical analysis

For statistical analysis, PASW/SPSS (Version 21) was used. Data were analyzed by two-way ANOVA for repeated measures (factors stimulus x group) or three-way ANOVA (factors genotype x treatment x gender), followed by a Bonferroni’s post-hoc analysis when appropriate. Statistical significance was set at p < 0.05. Overall statistics are shown in Table 1.

**Table 1:** Overall statistics for the behavioral data. Stimulus effect refers to both the empty cage and the *same* mouse (Fig 1A), to the *novel* and the *same* mouse/juvenile (Fig 1B/1C), and to the *novel* and the *same* object (Fig 2A and 2B).

	Stimulus effect	Stimulus x group effect
Social approach test (Fig 1A)	F(1,89) = 150.2; p<0.001*	F(7,89) = 2.49; p = 0.02*
Social approach test (Fig 1B)	F(1,89) = 85.53; p<0.001*	F(7,89) = 0.77; p = 0.61
Social discrimination test (Fig 1C)	F(1,40) = 48.11; p<0.001*	F(7,40) = 0.18; p = 0.99
Novel object recognition test (Fig 2A)	F(1,89) = 54.41; p<0.001*	F(7,89) = 0.95; p = 0.47
Object discrimination test (Fig 2B)	F(1,40) = 52.62; p<0.001*	F(7,40) = 0.92; p = 0.50
	Treatment effect	Genotype x gender effect
Novelty suppressed feeding (Fig 3)	F(1,85) = 29.11; p<0.001*	F(1,85) = 4.26; p = 0.04*

Group effect refers to each individual group, i.e. water-treated WT males = group 1; amitriptyline-treated WT males = group 2; water-treated t-ASM males = group 3; etc. Two-way ANOVA for repeated measures (factors stimulus x group) or three-way ANOVA (factors genotype x treatment x gender) followed by Bonferroni post-hoc test;

*p < 0.05.

## Results

### ASM overexpression impaired social preference without altering social memory in female, but not in male, mice

**Fig 1:**
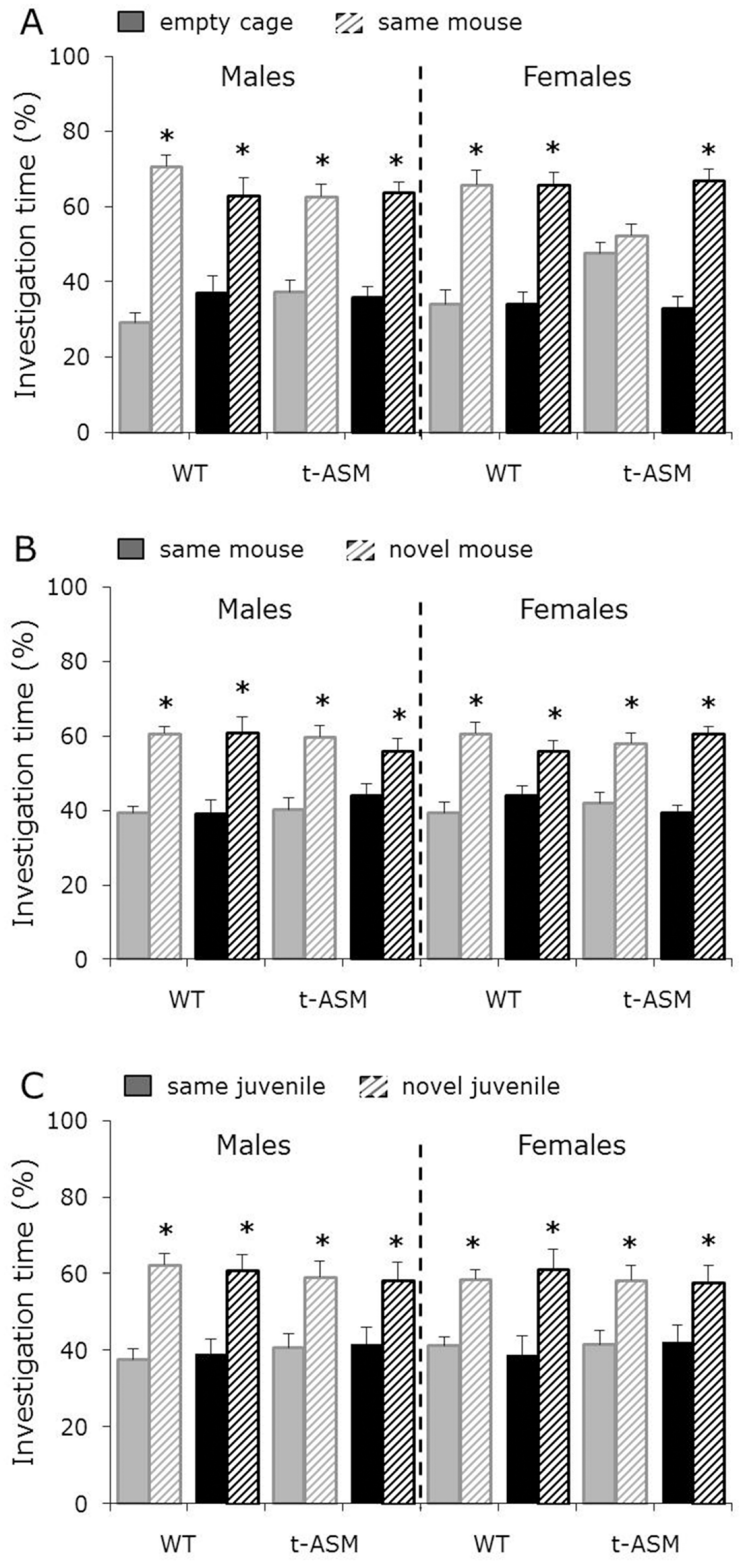
ASM overexpression impaired social preference (A) without altering preference for social novelty (B) or social memory (C) in female, but not in male, mice. Percentage investigation of the empty cage, the *same* and the *novel* mouse/juvenile during the first (A) and second (B) 5 min of the social approach test (n = 10–13 per group) and during the discrimination period of the social discrimination test (C; n = 6 per group). Mice were treated with amitriptyline (black bars) or water (grey bars) for 4 weeks before and during behavioral testing. Social preference is reflected by higher investigation time directed towards the *same* mouse versus the empty cage (A). Preference for social novelty (B) and social discrimination (C), as indicators of intact social memory, are reflected by higher investigation time directed towards the *novel* versus the *same* mouse/juvenile. Shown are means + SEM. * p<0.05 versus empty cage (A) or *same* mouse (B)/juvenile (C).

Males showed a higher investigation of the *same* mouse versus the empty cage during the first 5 min of the social approach test, independent of genotype and treatment, reflecting social preference. Whereas water- and amitriptyline-treated WT females showed social preference, water-treated t-ASM females showed lack of social preference, reflected by similar investigation of the *same* mouse and the empty cage. This lack of social preference could be prevented by amitriptyline treatment, resulting in reinstatement of social preference (p < 0.05 *same* mouse versus empty cage, Fig 1A). In addition, both males and females showed a higher investigation of the *novel* versus the *same* mouse during the second 5 min of the social approach test, independent of genotype and treatment, reflecting social recognition and preference for social novelty (p < 0.05, Fig 1B).

Males and females showed a higher investigation of the *novel* versus the *same* juvenile during the discrimination period of the social discrimination test, independent of genotype and treatment, reflecting social discrimination and intact social memory (p < 0.05, Fig 1C).

### ASM overexpression protected female mice against the detrimental effects of amitriptyline on non-social memory

**Fig 2:**
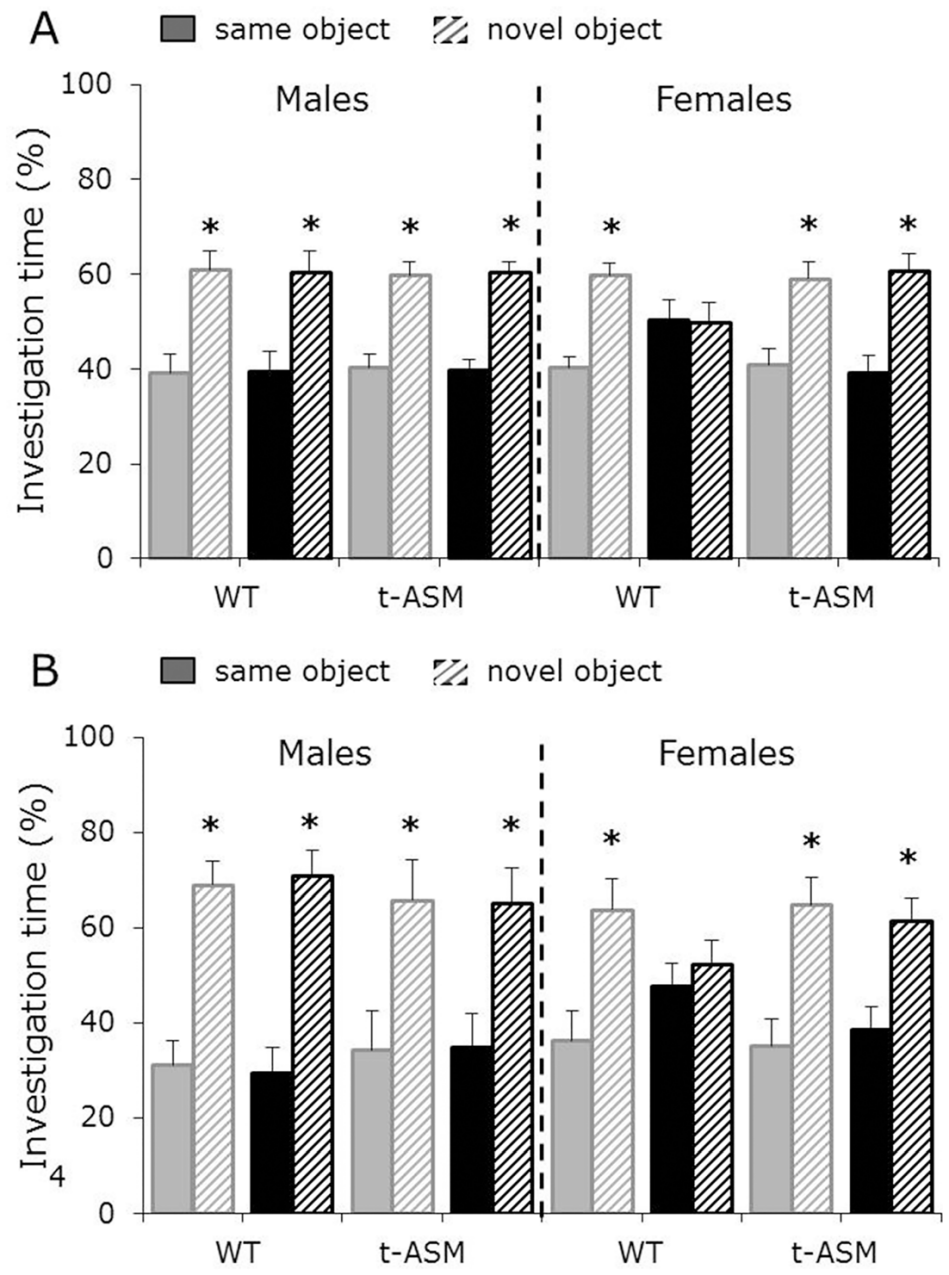
ASM overexpression protected females against the detrimental effects of amitriptyline on non-social memory performance. Percentage investigation of the *same* and the *novel* object during the second 5 min of the novel object recognition test (A; n = 10–13 per group) and during the discrimination period of the object discrimination test (B; n = 6 per group). Mice were treated with amitriptyline (black bars) or water (grey bars) for 4 weeks before and during behavioral testing. Object recognition (A) and object memory (B), as indicators of intact non-social memory, are reflected by higher investigation time directed towards the *novel* versus the *same* object. Shown are means + SEM. * p<0.05 versus *same* object.

Males showed a higher investigation of the *novel* versus the *same* object during the second 5 min of the novel object recognition test, independent of genotype and treatment, reflecting object recognition and preference for novelty. Whereas water-treated WT and t-ASM females showed intact object recognition, amitriptyline-treated WT, but not t-ASM, females showed impaired object recognition, reflected by similar investigation of the *same* and the *novel* object (p < 0.05, Fig 2A).

Males showed a higher investigation of the *novel* versus the *same* object during the discrimination period of the object discrimination test, independent of genotype and treatment, reflecting object discrimination and intact non-social memory. Whereas water-treated WT and t-ASM females showed intact object discrimination, amitriptyline-treated WT, but not t-ASM, females showed impaired object discrimination, reflected by similar investigation of the *same* and the *novel* object (p < 0.05, Fig 2B).

### ASM overexpression increases depressive- and anxiety-like behavior in female, but not in male, mice

**Fig 3:**
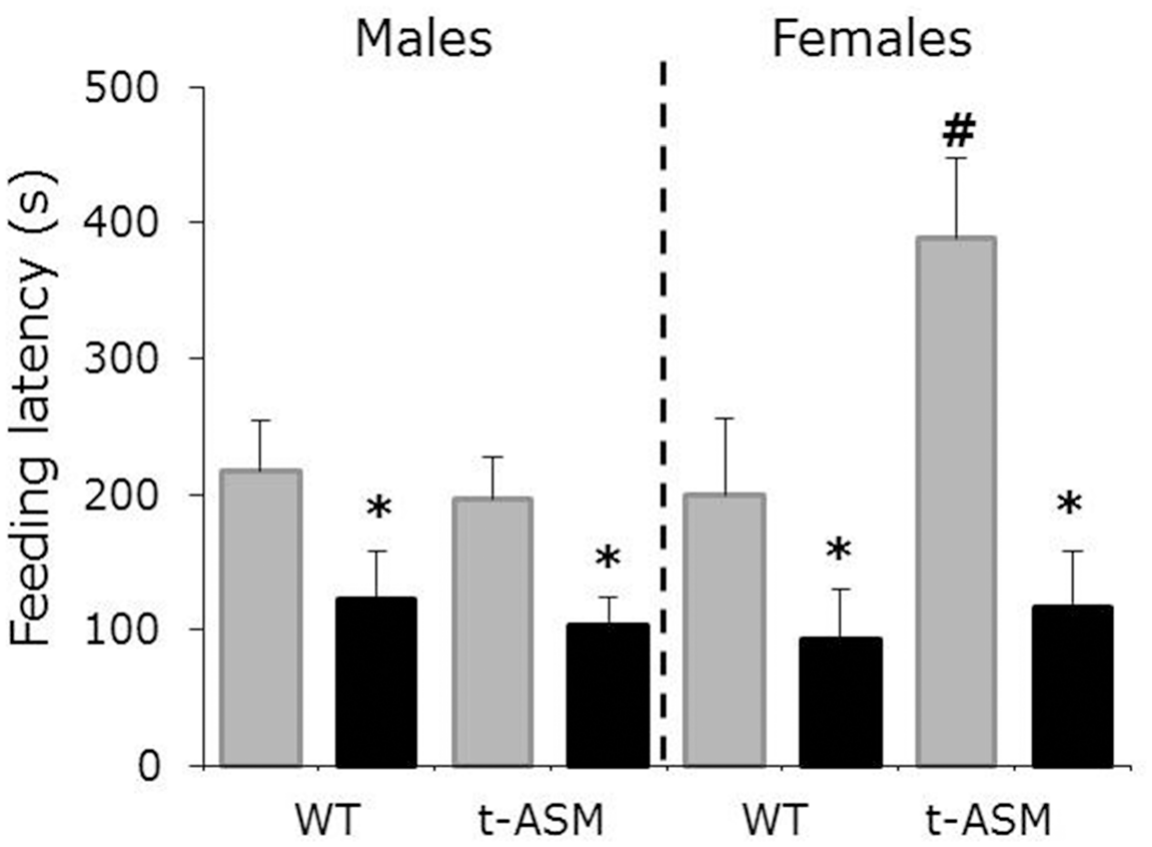
ASM overexpression increased depressive- and anxiety-like behavior in female, but not in male, mice. Feeding latency in a novel arena after a fasting period of 24 h. Mice were treated with amitriptyline (black bars) or water (grey bars) for 4 weeks before and during behavioral testing (n = 10–13 per group). Shown are means + SEM. p<0.05 * versus water-treated mice; # versus WT mice.

Water-treated female, but not male, t-ASM mice showed an increased feeding latency after a fasting period of 24 h, reflecting increased depressive- and anxiety-like behavior (p < 0.05 versus WT, Fig 3). Amitriptyline reduced feeding latency in both male and female mice, independent of genotype, reflecting decreased depressive- and anxiety-like behavior (p < 0.05 versus water-treated mice, Fig 3).

## Discussion

This study demonstrates that ASM overexpression alters the behavior of mice in a sex-dependent manner. In more detail, we could show that ASM overexpression impaired social preference and induced a depressive- and anxiogenic-like behavior in female, but not in male, mice without altering the preference for social novelty or social memory abilities in either sex. Furthermore, ASM overexpression had a protective role against the detrimental effects of amitriptyline on female, but not on male, non-social (object) memory.

We have previously shown a depressive- and anxiogenic-like phenotype in t-ASM mice in the novelty-suppressed feeding paradigm and in the open field test, which was normalized by amitriptyline treatment [9]. The present study confirmed this effect and extended these findings by demonstrating that only female t-ASM mice showed this phenotype. Although Gulbins et al. [9] have mainly used female t-ASM mice they made no distinction between sexes in their study.

According to our hypothesis, t-ASM mice showed a lack of social preference, indicating deficits in social functioning. Similar to the depressive- and anxiogenic-like phenotype, these deficits were only seen in female t-ASM mice, and could be normalized by amitriptyline treatment. A possible explanation for these sex-dependent effects might be the modulatory effect of estrogens on ceramide synthesis. Ceramide can be generated either through the hydrolysis of sphingomyelin, reaction catalyzed by the enzymes sphingomyelinases, including ASM and the neutral sphingomyelinases 2–4, through de novo synthesis, reaction catalysed by several enzymes, including ceramide synthases (CerS), and through a salvage pathway. Wegner et al. [16] have shown that 17β-estradiol increased the activity of ceramide synthases CerS4 and CerS5 in human breast cancer cells. It might be therefore possible that female t-ASM mice have higher ceramide concentrations than male t-ASM mice due to increased de novo synthesis, which in turn lead to a depressive- and anxiogenic-like phenotype reflected as increased feeding latency in the novelty-suppressed feeding paradigm and lack of social preference in the social approach test. In support of this hypothesis, Ishikawa et al. [17] have shown that serum from healthy women contained higher levels of 42:1 ceramide metabolites than serum from healthy men. However, it remains to be verified in future studies whether female t-ASM mice show higher ceramide concentrations than male t-ASM mice.

Contrary to our hypothesis, t-ASM mice did not show any signs of memory impairment, neither in social, nor in non-social (object) memory. In more detail, both male and female t-ASM mice showed unaltered preference for social novelty and social discrimination, indicating intact short-term social memory abilities. Similarly, both male and female t-ASM mice showed unaltered novel object recognition and object discrimination, indicating intact short-term non-social memory abilities. Interestingly, amitriptyline treatment impaired both novel object recognition and object discrimination in female WT, but not t-ASM, mice, indicating that ASM overexpression protected female mice against the detrimental effects of amitriptyline on non-social memory. Amitriptyline has been previously shown to impair learning tasks in rodents after acute [18–21] and chronic [21,22] administration. Although Everss et al. [21] have shown that chronically-administered amitriptyline impaired learning in both male and female mice, piracetam, a cyclic derivative of GABA which was shown to counteract the amnesic effects of a variety of drugs in rodents [23–25], counteracted the effects of amitriptyline in male, but not in female, mice suggesting that the effects of amitriptyline on memory performance might be more severe in females. Orsetti et al. [22] have shown that chronic administration of amitriptyline over 4 weeks via i.p. injections exerted a dose-dependent effect on object discrimination in male Wistar rats, i.e. it impaired object discrimination at 5 mg/kg, but did not affect object discrimination at 2 mg/kg. It might be therefore possible for higher doses of amitriptyline to impair object memory in male WT mice as well.

Interestingly, female t-ASM mice were protected against the detrimental effects of amitriptyline on non-social memory performance. We have previously shown that amitriptyline inhibited the activity of ASM in the hippocampus in both WT and t-ASM mice, thereby decreasing ceramide concentration [9]. Tabatadze et al. [26] have shown that the spatial and episodic memory of mice was impaired after inhibition of neutral sphingomyelinase-2, an enzyme that rapidly generates ceramide and seems to function upstream of ASM [27]. It might be therefore possible that the impairments in non-social memory observed in female WT mice after amitriptyline treatment are due to a decrease in ceramide concentration. In this case, female t-ASM mice might be protected against these effects of amitriptyline due to their higher basal ceramide concentration resulting from the increased ASM activity.

Taken together, we have shown that ASM overexpression results in sex-dependent effects on social, depressive- and anxiety-like behavior. Despite these negative effects on behavior, ASM overexpression protected female mice against the detrimental effects of amitriptyline on non-social memory performance. This study suggests that female t-ASM mice represent a model of depression with comorbid anxiety and social deficits, without impairments in social and non-social memory.
